# Risk of stroke or systemic embolism in patients with degenerative mitral stenosis with or without atrial fibrillation: A cohort study

**DOI:** 10.1016/j.ijcha.2022.101126

**Published:** 2022-10-07

**Authors:** Daniel Kjærgaard Steiner, Peter Søgaard, Martin Jensen, Torben Bjerregaard Larsen, Gregory Yoke Hong Lip, Peter Brønnum Nielsen

**Affiliations:** a Aalborg Thrombosis Research Unit, Department of Clinical Medicine, Faculty of Health, Aalborg University, Aalborg, Denmark; b Department of Cardiology, Aalborg University Hospital, Aalborg, Denmark; c Liverpool Centre for Cardiovascular Science, University of Liverpool and Liverpool Heart & Chest Hospital, Liverpool, UK

**Keywords:** Mitral stenosis, Valvular heart disease, Stroke, Atrial fibrillation, Anticoagulation, DMS, degenerative mitral stenosis, DOAC, direct oral anticoagulants, MAC, mitral annular calcification, MS, mitral stenosis, NOAC, non-vitamin K-antagonist oral anticoagulants, OAC, oral anticoagulants, VKA, vitamin K-antagonist

## Abstract

**Background:**

Atrial fibrillation (AF) is associated with an increased risk of stroke and the combination of AF and mitral stenosis (MS) is associated with a higher risk. In developed nations, degenerative mitral stenosis (DMS) constitutes a sizeable proportion of patients with MS. Current international guidelines do not offer recommendations regarding anticoagulation in these patients. The objective of this study was to describe the incidence of stroke or systemic embolism in patients with DMS with or without prevalent AF.

**Methods:**

A cohort study of DMS patients from 1997 to 2018, using data from the Danish health registries. The cohort was stratified based on AF prevalence and prior ischemic stroke. The primary outcome was a diagnosis of ischemic stroke or systemic embolism after 1 year of follow-up from time of DMS diagnosis.

**Results:**

The study included 1162 patients with DMS, of which 421 had prevalent AF. The incidence rate of stroke or systemic embolism after 1 year of follow-up was highest in the DMS without AF group (7.58 vs. 6.63 per 100 person-years). In both groups, DMS without AF and DMS with AF, the incidence rate was highest in patients with prior thromboembolic events (29.61 vs. 5.15 and 19.53 vs. 5.15, respectively).

**Conclusions:**

The incidence rate of stroke or systemic embolism was highest in DMS patients without AF. Current Danish guidelines recommend DMS patients should be treated with anticoagulation only with concurrent AF, yet our results call for additional research to establish if DMS patients without AF could benefit from stroke prevention therapy.

## 1 Introduction

Atrial fibrillation (AF) is associated with an increased risk of stroke and can occur in isolation or concurrently with various pathologies such as mitral stenosis (MS) [1]. Degenerative mitral stenosis (DMS) is the most prevalent type of non-rheumatic MS, often related to mitral annular calcification (MAC) [2], [3], [4], [5]. DMS is commonly found in patients with cardiovascular risk factors, particularly among females, and the frequency is expected to rise in an aging population where these risk factors accumulate [6], [7], [8]. The prevalence of DMS has previously been estimated between 0.19% and 0.22% [6], [9]. In developed nations, DMS constitutes a sizeable proportion of patients with MS: for example, in the Euro Heart Survey DMS was shown by echocardiographic assessment to account for 12.5% of all cases out of 336 patients with MS, with the majority presenting with severe and symptomatic disease [10]. In a more recent study of 50 patients with MAC assessed by echocardiography, DMS was found in 26% [11]. MAC has previously been associated with an increased risk of stroke, even in the absence of AF [12], [13].

Patients with moderate-to-severe MS were excluded from the pivotal NOAC-trials because of a particularly high risk for thromboembolism [14], [15]. A consensus statement from the European Heart Rhythm Association states that patients with AF and any degree of rheumatic MS and moderate-to-severe non-rheumatic MS should be treated with vitamin K-antagonists (VKA), until more data are available [14]. The most recent ACC/AHA-, ESC/EACTS- and APHRS-guidelines do not offer any recommendations regarding anticoagulation in patients with DMS [16], [17], [18].

Results from previous prospective studies, estimating the risk of stroke or systemic embolism in MS patients with concurrent AF, were summarized by Karthikeyan et al. [15]. The incidence rates reported were in the range of 0.4 to 3.9 per 100 person-years, after up to 4.5 years of follow-up, however these studies included patients with MS of both rheumatic and non-rheumatic etiology [19], [20], [21]. Also, most of the data available on stroke-risk in MS and AF come from retrospective studies that are several decades old on patients with rheumatic MS [15], [22].

The objective of this study was to describe the incidence of ischemic stroke or systemic embolism in an unselected nationwide cohort of patients with DMS with or without prevalent AF.

## 2 Methods

### 2.1 Data source and study population

This was a registry-based cohort study using data from the Danish health registries, which apply a unique personal identification number assigned to all Danish residents at birth or emigration. The Danish National Patient Register records all information on diagnostic procedures and treatment in Danish hospitals, community or privately owned [23]. The Civil Registration System provides information on sex, age, vital status, and emigration [24], [25]. The Danish National Prescription Registry collects information on claimed prescriptions [26]. All data used in the study was retrieved from these databases and made available by the Danish Health Data Authority.

**Supplementary figure 1:**
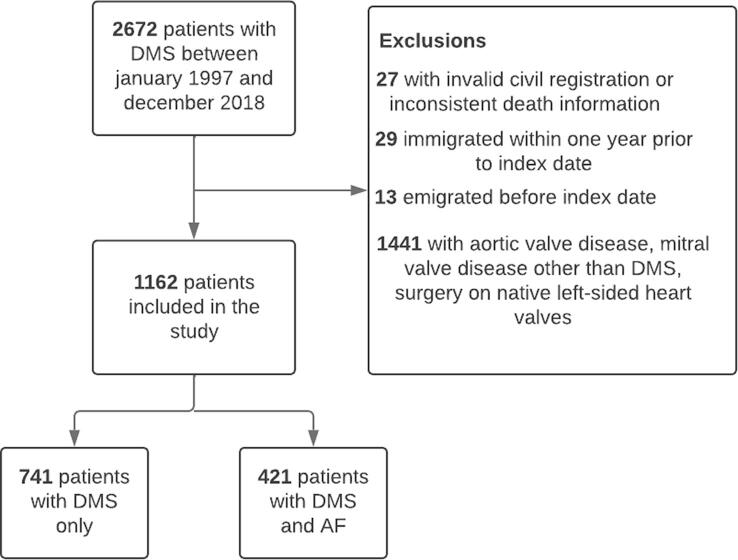
Flowchart of the cohort.

The study population included patients diagnosed with mitral stenosis on a non-rheumatic basis (i.e., degenerative MS) between January 1, 1997, and December 31, 2018. The registries were screened for patients with a record of non-rheumatic MS according to the ICD-10 (International Classification of Diseases, 10th revision). Patients with mitral valve prolapse and rheumatic- and congenital mitral valve disease were excluded from the study. Patients with previous surgery on native left-sided valves and/or a diagnosis of aortic valve disease were excluded. See Supplementary Fig. 1 and Supplementary Table 1 (appendix section) for details on patient selection.

Baseline (index date) was defined as the date of an incident DMS diagnosis, and the cohort was stratified based on a record of prevalent AF and categorized as “DMS without AF” and “DMS with AF”.

### 2.2 Study variables

Variables included in the study were age, female sex, hypertension, prior thromboembolic events, dyslipidemia, obesity, cardiomyopathy, diabetes mellitus, congestive heart failure, ischemic heart disease, chronic kidney disease, peripheral artery disease, dialysis due to chronic renal failure, coronary revascularization procedures (percutaneous coronary intervention or coronary artery bypass graft), ablation therapy and pacemaker implantation. The CHA_2_DS_2_-VASc score, denoting congestive heart failure, hypertension, age ≥75 years, diabetes mellitus, prior stroke, systemic embolism or transient ischemic attack, vascular disease, age 65–74 years, and sex category was also included. The National Prescription Registry was used to assess claimed prescriptions for medical therapy based on pharmacy redemptions within the last year before the index date by using the Anatomical Therapeutic Chemical (ATC)-codes (see Supplementary Table 1).

### 2.3 Follow-up and outcomes

Patients were followed in the Danish National Patient Register for up to 1 year after baseline for a record of ischemic stroke or systemic embolism. An outcome of systemic embolism included emboli in the peripheral arteries of the upper- and lower extremity as well as in the aorta, iliac-, renal-, splenic- and mesenteric arteries. All-cause mortality was included as a secondary outcome. An analysis stratifying event rates by CHA_2_DS_2_-VASc score was also performed as “No treatment indication” comprising males with a score of 0, and females with a score of 1; “Consider treatment” comprised of males with a score of 1, and females with a score of 2; “Recommend treatment” comprising males with a score of ≥2, and females with a score of ≥3. Last, an analysis stratifying the cohort by prior thromboembolic events was also conducted.

### 2.4 Statistics

Baseline characteristics were ascertained at the time of DMS diagnosis and stratified by AF diagnosis. Percentages and number count were provided for categorical data, and continuous data are presented with means and standard deviations (SD). Investigations of outcomes were performed by means of time-to-event analyses. Patients were followed for up to 1 year from the time of DMS diagnosis to an outcome of interest, study end (December 31, 2018), emigration, or death – whichever came first. Risks of outcomes over time were depicted with cumulative incidence curves using the Aalen-Johansen estimate for competing risk of death. Time-to-event analyses and incidence rates were calculated as number of events per 100 person-years.

Three sensitivity analyses were also performed. First, for the primary outcome, with and without stratification by prior thromboembolic events, the analysis was performed using a 10-day quarantine period following the index date, hereby postponing the counting of days at risk. This was done to reduce the risk of carry-over diagnoses, i.e., an outcome diagnosis erroneously given to patients with a prior thromboembolic event very close to the index date, leading to an exaggerated stroke rate [27]. Second, an analysis on the incidence of AF after the index date in the “DMS without AF” cohort was performed to evaluate the classification of AF status at baseline. Third, event rates for the primary outcomes were calculated for patients not treated with oral anticoagulants (OAC) at index date, with censoring of person-time occurring when treatment with oral anticoagulants was initiated during follow-up. This was done to get an indication of the natural history for stroke in this subgroup.

Data management and analyses were performed using SAS 9.3 software (SAS Institute, Cary, North Carolina) and STATA/MP version 16 software (StataCorp LP, College Station, Texas).

## 3 Results

A total of 2672 patients with DMS were identified, and after exclusions, 1162 patients (71% females) with DMS were included in the study, of which 421 (36.2%) had prevalent AF. Patients with AF were older (mean 73 vs. 67 years), and had a higher prevalence of comorbidity, in particular congestive heart failure (41.1% vs. 21.9%), ischemic heart disease (32.8% vs. 24.8%) and cardiomyopathy (4.0% vs. 1.8%). The higher prevalence of comorbidity in the DMS with AF group was also reflected by a higher mean CHA_2_DS_2_-VASc score (3.9 vs. 3.3). The number of patients on oral anticoagulation treatment with VKAs was more than three times higher in the DMS with AF group (49.2% vs. 15.9%). The prevalence of prior stroke or systemic embolism was 12.1% in the DMS with AF group and 11.1% in the DMS without AF group. See Table 1 for full details.

**Table 1:** Baseline characteristics of patients with DMS stratified by AF status.

	Total (n = 1162)	DMS without AF (n = 741)	DMS with AF (n = 421)
Age in years, mean (SD)	69.9 (14.8)	67.9 (16.1)	73.4 (11.6)
Female sex, % (n)	71.2 (827)	70.0 (519)	73.2 (308)
Hypertension, % (n)	48.4 (562)	45.6 (338)	53.2 (224)
Prior stroke or systemic embolism, % (n)	11.4 (133)	11.1 (82)	12.1 (51)
Dyslipidaemia, % (n)	10.5 (122)	11.1 (82)	9.5 (40)
Obesity, % (n)	5.2 (61)	5.4 (40)	5.0 (21)
Cardiomyopathy, % (n)	2.6 (30)	1.8 (13)	4.0 (17)
Diabetes mellitus, % (n)	17.5 (203)	17.5 (130)	17.3 (73)
Congestive heart failure, % (n)	28.8 (335)	21.9 (162)	41.1 (173)
IHD, % (n)	27.7 (322)	24.8 (184)	32.8 (138)
CKD, % (n)	7.1 (82)	7.3 (54)	6.7 (28)
PAD, % (n)	7.1 (83)	6.6 (49)	8.1 (34)
CHA_2_DS_2_-VASc, mean (SD)	3.5 (1.9)	3.3 (1.9)	3.9 (1.8)
CHA_2_DS_2_-VASc score, % (n)			
Males 0, females 1	12.9 (150)	16.5 (122)	6.7 (28)
Males 1, females 2	13.6 (158)	14.4 (107)	12.1 (51)
Males ≥ 2, females ≥ 3	73.5 (854)	69.1 (512)	81.2 (342)
Coronary revascularization (PCI/CABG), % (n)	4.0 (46)	3.5 (26)	4.8 (20)
Pacemaker, % (n)	2.6 (20)	1.9 (14)	3.8 (16)
Ablation therapy, % (n)	– (<5)	– (<5)	– (<5)
Dialysis, % (n)	2.1 (24)	2.2 (16)	1.9 (8)
Pharmacological therapy, % (n)			
VKA	28.0 (325)	15.9 (118)	49.2 (207)
Aspirin	36.0 (418)	35.0 (259)	37.8 (159)
Thienopyridines	5.0 (58)	5.5 (41)	4.0 (17)
CCB	28.5 (331)	26.6 (197)	31.8 (134)
ACEI/ARB	38.1 (443)	38.1 (282)	38.2 (161)
Beta-blocker	29.3 (340)	22.1 (164)	41.8 (176)
Diuretics	66.4 (771)	60.9 (451)	76.0 (320)
Statins	24.6 (286)	25.6 (190)	22.8 (96)

n = number of patients, SD = standard deviation, AF = atrial fibrillation, DMS = degenerative mitral stenosis, IHD = ischemic heart disease, CKD = chronic kidney disease, PAD = peripheral arterial disease, PCI = percutaneous coronary intervention, CABG = coronary artery bypass graft, VKA = vitamin K-antagonist (warfarin and phenprocoumon were included), CCB = calcium-channel blocker, ACEI = angiotensin-converting enzyme inhibitor, ARB = angiotensin receptor blocker. Thienopyridines included were clopidogrel, ticagrelor, and prasugrel.

### 3.1 Incidence rates and cumulative incidence

**Fig. 1:**
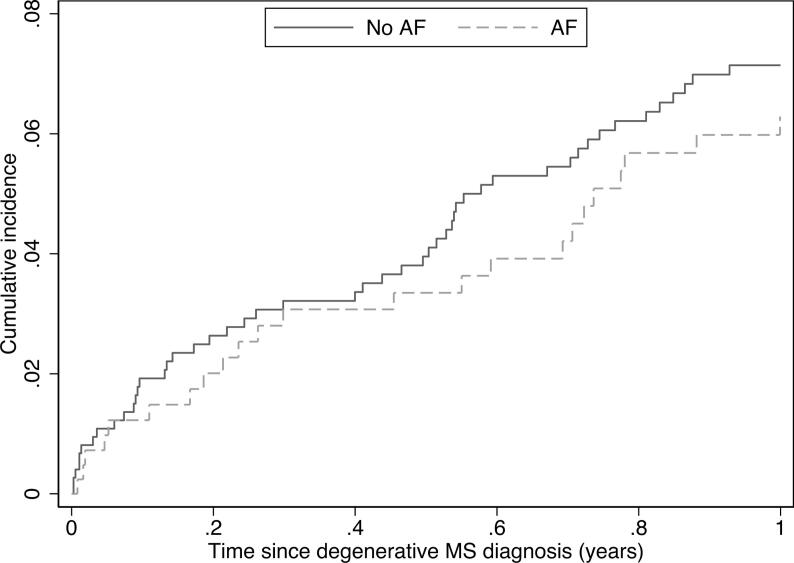
DMS = degenerative mitral stenosis, AF = atrial fibrillation. 1-year cumulative incidence of ischemic stroke or systemic embolism in patients with DMS stratified by AF status.

**Supplementary figure 2:**
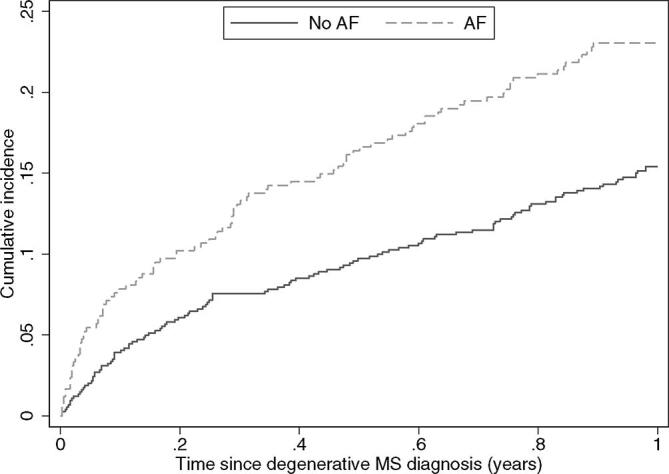
DMS = degenerative mitral stenosis, AF = atrial fibrillation. 1-year cumulative incidence of all-cause mortality in patients with DMS stratified by AF status.

Table 2 presents the number of events and incidence rates per 100 person-years (95% CI) for stroke or systemic embolism and all-cause mortality after 1 year of follow-up. A total of 72 events were observed. The incidence rate per 100 person-years of stroke or systemic embolism was highest in the DMS without AF group (7.58 vs. 6.63). All-cause mortality at 1 year of follow-up was highest in the DMS with AF group: incidence rate 27.28 vs. 17.0. The cumulative incidences of stroke or systemic embolism and all-cause mortality after 1 year of follow-up, in patients with DMS stratified by AF status, are depicted in Fig. 1 and Supplementary Fig. 2, respectively.

**Table 2:** Events and incidence rates per 100 person-years (95% CI) for ischemic stroke or systemic embolism and all-cause mortality after 1 year of follow-up.

	Ischemic stroke or systemic embolism			All-cause mortality	
	No. of events		Incidence rate (95% CI)	No. of events	Incidence rate (95% CI)
DMS without AF group (n = 741)	49		7.58 (5.73–10.03)	114	17.0 (14.15–20.43)
DMS with AF group (n = 421)	23		6.63 (4.41–9.98)	97	27.28 (22.35–33.28)

AF = atrial fibrillation, CI = confidence interval, DMS = degenerative mitral stenosis, n = number of patients.

Table 3 presents the number of events and incidence rates for ischemic stroke or systemic embolism and all-cause mortality after 1 year of follow-up stratified by prior thromboembolic event. The incidence rate of stroke or systemic embolism after 1 year of follow-up was highest in patients with prior thromboembolic events, regardless of AF status. In the DMS without AF group, the incidence rate was 29.61 in patients with a prior thromboembolic event vs. 5.15 in patients with no prior thromboembolic event. Likewise, in the DMS with AF group, the incidence rate was 19.53 in patients with a prior thromboembolic event vs. 5.15 in patients without a prior thromboembolic event. In the DMS without AF group, the incidence rate for all-cause mortality at 1 year of follow-up, was 10.39 in patients with prior thromboembolic events vs. 17.86 in patients with no prior thromboembolic events. In the DMS with AF group, the incidence rate was highest in patients with prior thromboembolic events (44.25 vs. 25.22).

**Table 3:** Events and incidence rates per 100 person-years (95% CI) for ischemic stroke or systemic embolism and all-cause mortality after 1 year of follow-up stratified by prior thromboembolic event.

	Ischemic stroke or systemic embolism		All-cause mortality	
	No. of events	Incidence rate (95% CI)	No. of events	Incidence rate (95% CI)
DMS without AF group				
No prior thromboembolic event (n = 659)	30	5.15 (3.60–7.37)	106	17.86 (14.76–21.60)
Prior thromboembolic event (n = 82)	19	29.61 (18.88–46.41)	8	10.39 (5.20–20.78)
DMS with AF group				
No prior thromboembolic event (n = 370)	16	5.15 (3.15–8.40)	80	25.22 (20.26–31.40)
Prior thromboembolic event (n = 51)	7	19.53 (9.31–40.97)	17	44.25 (27.51–71.18)

AF = atrial fibrillation, CI = confidence interval, DMS = degenerative mitral stenosis, n = number of patients.

In Table 4, the number of events and incidence rates for ischemic stroke or systemic embolism after 1 year of follow-up stratified by CHA_2_DS_2_-VASc score are presented. Incidence rates of the main outcome were highest in the “recommend treatment” group: 8.74 in the DMS without AF group vs. 8.10 in the DMS with AF group. In the “consider treatment” group, incidences rates were 6.17 in the DMS without AF group vs. 2.07 in the DMS with AF group. In the “no treatment indication” group, incidences rates were 4.39 in the DMS without AF group vs. 0.00 in the DMS with AF group.

**Table 4:** Events and incidence rates per 100 person-years (95% CI) for ischemic stroke or systemic embolism and all-cause mortality after 1 year of follow-up stratified by CHA_2_DS_2_-VASc score.

	Ischemic stroke or systemic embolism	
	No. of events	Incidence rate (95% CI)
DMS without AF group		
CHA_2_DS_2_-VASc “no treatment” group (n = 122)	5	4.39 (1.83–10.54)
CHA_2_DS_2_-VASc “consider treatment” group (n = 107)	6	6.17 (2.77–13.73)
CHA_2_DS_2_-VASc “recommend treatment” group (n = 512)	38	8.74 (6.36–12.01)
DMS with AF group		
CHA_2_DS_2_-VASc “no treatment” group (n = 28)	<5	0.00
CHA_2_DS_2_-VASc “consider treatment” group (n = 51)	<5	2.07 (0.29–14.72)
CHA_2_DS_2_-VASc “recommend treatment” group (n = 342)	22	8.10 (5.34–12.31)

AF = atrial fibrillation, CI = confidence interval, DMS = degenerative mitral stenosis, n = number of patients. Event counts < 5 are masked as required by Danish registry data legislations.

### 3.2 Sensitivity analyses

Supplementary Table 2 and Supplementary Table 3 presents the sensitivity analysis using a 10-day quarantine period on the main outcome analysis. The incidence rate of stroke or systemic embolism after 1 year of follow-up was highest in the DMS without AF group (6.65 vs. 5.77). Regardless of AF, patients with prior thromboembolic events had the highest incidence rates for ischemic stroke or systemic embolism after 1 year of follow-up. In the DMS without AF group, the incidence rate was 24.93 in patients with a prior thromboembolic event vs. 4.64 in patients with no prior thromboembolic event. In the DMS with AF group, the incidence rate was 13.95 in patients with a prior thromboembolic event vs. 4.82 in patients without a prior thromboembolic event.

**Supplementary figure 3:**
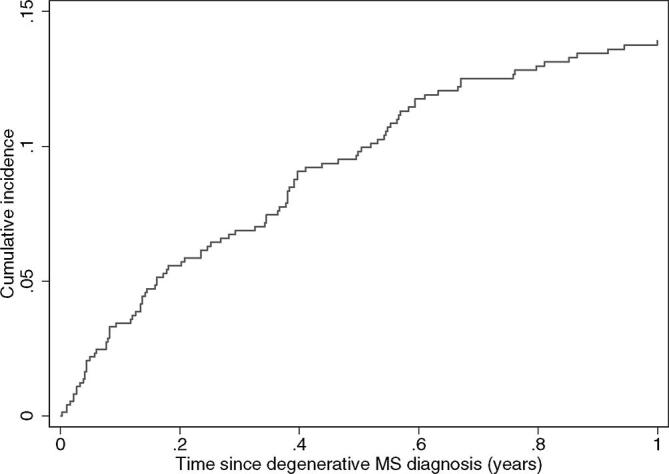
DMS = degenerative mitral stenosis, AF = atrial fibrillation. 1-year cumulative incidence of AF in DMS patients without AF at baseline.

The incidence rate of developing AF after index date among patients with DMS was 15.66 after 1 year of follow-up (see Supplementary Table 4). The 1-year cumulative incidence is depicted in Supplementary Fig. 3.

Supplementary Table 5 presents the subgroup analysis with event rates after 1 year of follow-up for patients not treated with OACs at index date, censored for when oral anticoagulants were started during follow-up. The incidence rate for stroke or systemic embolism was 7.30 in this subgroup.

## 4 Discussion

In this study of Danish DMS patients with or without AF, our principal finding was a 1-year incidence rate of ischemic stroke or systemic embolism of 7.58 and 6.63 in patients with DMS without AF and with AF, respectively. Second, when stratifying the cohort by prior thromboembolic events, the results show markedly higher incidence rates of stroke or systemic embolism in patients with a prior event, regardless of AF status. Third, the number of patients on oral anticoagulation treatment with VKAs was three times higher in the DMS with AF group.

Rheumatic MS is a well understood disease, but much less is known about DMS [28]. Patients with significant MS were excluded from the NOAC-trials and current Danish guidelines recommend that patients with MS (regardless of etiology) should be treated with VKAs, but not DOAC, and only in the presence of concurrent AF, a prior thromboembolic event or thrombus formation in the left atrial appendage, or a severely dilated left atrium [29]. The current ESC/EACTS-, ACC/AHA- and APHRS-guidelines do not offer any recommendations on treatment with anticoagulants in patients with DMS, but offer the same recommendations as stated above in patients with rheumatic MS [16], [17], [18].

De Catarina et al. [30] discussed the use of anticoagulation in MS patients, and state that even though these patients are considered the highest risk for thromboembolism, “there are no reasons to suggest a differential response to various anticoagulants”. Furthermore, a 2019, non-randomized study by Kim et al. [1] assessing the efficacy of prescribed off-label DOAC vs. conventional warfarin treatment in a population of patients with MS (rheumatic and non-rheumatic etiology) and AF, reported that stroke or systemic embolism occurred at a rate of 2.22% per year in the DOAC-group, and 4.19% per year in the warfarin group. In the present study, we observed an incidence rate of stroke or systemic embolism of 7.58 in patients with DMS without AF after 1 year of follow-up, and 15.9% percent of patients in this group were treated with VKAs. Albeit a limited sample, our results could signal that patients with DMS without prevalent AF, might benefit from anticoagulation in terms of stroke prevention. The much higher number of patients on VKAs in the DMS with AF group (49.2% vs. 15.9%) in this study, likely explains the lower incidence rate of stroke or systemic embolism observed in patients with prevalent AF. Our subgroup analysis in the non-OAC group showed an incidence rate of stroke or systemic embolism of 7.30 after 1 year of follow-up, indicating that DMS patients not treated with OACs are at a significant risk for thromboembolism, corroborating the hypothesis that this population might benefit from anticoagulation.

Indeed, it is of interest to evaluate the efficacy of DOACs in MS patients, whether on a rheumatic- or non-rheumatic basis, due to the advantage of not needing to monitor treatment. Currently, a randomized controlled trial evaluating the efficacy of rivaroxaban vs. warfarin in patients with rheumatic mitral stenosis and AF, is in progress [31].

Of note, the sensitivity analysis with a 10-day quarantine period after index date, showed lower incidence rates of the main outcome after 1 year of follow-up in both groups. Quarantine periods can be applied in registry-based studies and may reduce overestimation of the outcome by removing carry-over diagnoses erroneously given to patients. Hence the results of the main outcome analysis with a 10-day quarantine period, may represent a more accurate estimate, but could also lead to patients with a true recurrent stroke being excluded from the study [27].

### 4.1 Limitations

Our study has limitations that must be acknowledged. We used nationwide registries spanning 21 years of data but only identified a relatively limited number of patients diagnosed with DMS. MAC and subsequent development of DMS is not a treatable condition, thus possibly leading to underreporting by physicians not coding the finding in accordance with ICD-10. Identifying DMS patients might be better accomplished by other means, such as evaluating echocardiographic features like mitral valve area, which is typically used to grade the severity of MS [16]. Since we had no information on valve characteristics, we could not ascertain the severity of mitral stenosis, but only report if a patient had DMS or not. Furthermore, we had no information on INR and hence the efficacy of anticoagulation in this cohort. Unfortunately, it was not possible to distinguish between subtypes of atrial fibrillation nor determine the duration of an episode, using the Danish national health registries. Yet, current international guidelines do not distinguish between subtypes of atrial fibrillation with regards to treatment. Denmark follows international guideline recommendations suggesting a 12-lead ECG or Holter monitoring to confirm an atrial fibrillation diagnosis detected from (e.g.) opportunistic screening and palpation. Thus, we still believe our manuscript is of clinical relevance while noting this limitation. The results of this study were contingent on the coding of DMS diagnoses in the Danish health registries. No validation studies on the DMS diagnosis in the Danish National Patient Register exists. Different studies have ascertained positive predictive values for various discharge diagnoses in the Danish National Patient Register. The positive predictive value of a stroke discharge diagnosis has previously been found to be 69.3% in one study [32]. In another study, a diagnosis of stroke was found to have a positive predictive value between 74% and 97% [33]. In a third study, a diagnosis of AF was found to have a positive predictive value of 92.6% [34].

## 5 Conclusion

The 1-year incidence rate of stroke or systemic embolism was highest in DMS patients without AF, and patients with a prior thromboembolic event had a higher incidence of this outcome compared to those without a prior event, regardless of AF status. As current guidelines recommend that DMS patients should be treated with anticoagulation only with concurrent AF, our results call for additional studies to establish if DMS patients without AF could benefit from stroke prevention therapy.

## Disclosures

Professor Lip: Consultant and speaker for BMS/Pfizer, Boehringer Ingelheim and Daiichi- Sankyo. No fees are received personally. Professor Larsen: Investigator for Janssen Scientific Affairs, LLC, and Boehringer Ingelheim. Speaker for Bayer, BMS/Pfizer, Janssen Pharmaceuticals, Takeda, Roche Diagnostics, and Boehringer Ingelheim. Mr. Nielsen: Speaking fees Daiichi-Sankyo, consulting fees from Bayer and Daiichi-Sankyo, and grant support from BMS/Pfizer and Daiichi-Sankyo.

## Declaration of Competing Interest

The authors declare that they have no known competing financial interests or personal relationships that could have appeared to influence the work reported in this paper.

## References

[1] Kim J.Y., Kim S.-H., Myong J.-P., Kim Y.R., Kim T.-S., Kim J.-H., Jang S.-W., Oh Y.-S., Lee M.Y., Rho T.-H. (2019). Outcomes of direct oral anticoagulants in patients with mitral stenosis. J. Am. Coll. Cardiol..

[2] Abramowitz Y., Jilaihawi H., Chakravarty T., Mack M.J., Makkar R.R. (2015). Mitral annulus calcification. J. Am. Coll. Cardiol..

[3] Banovic M., DaCosta M. (2019). Degenerative mitral stenosis: from pathophysiology to challenging interventional treatment. Curr. Probl. Cardiol..

[4] Savage D.D., Garrison R.J., Castelli W.P., McNamara P.M., Anderson S.J., Kannel W.B., Feinleib M. (1983). Prevalence of submitral (anular) calcium and its correlates in a general population-based sample (the Framingham study). Am. J. Cardiol..

[5] Fulkerson P.K., Beaver B.M., Auseon J.C., Graber H.L. (1979). Calcification of the mitral annulus. Etiology, clinical associations, complications and therapy. Am. J. Med..

[6] Ukita Y., Yuda S., Sugio H., Yonezawa A., Takayanagi Y., Masuda-Yamamoto H., Tanaka-Saito N., Ohnishi H., Miura T. (2016). Prevalence and clinical characteristics of degenerative mitral stenosis. J. Cardiol..

[7] Al‐Taweel A., Almahmoud M.F., Khairandish Y., Ahmad M. (2019). Degenerative mitral valve stenosis: diagnosis and management. Echocardiography..

[8] Sud K., Agarwal S., Parashar A., Raza M.Q., Patel K., Min D., Rodriguez L.L., Krishnaswamy A., Mick S.L., Gillinov A.M., Tuzcu E.M., Kapadia S.R. (2016). Degenerative mitral stenosis: unmet need for percutaneous interventions. Circulation.

[9] Akram M.R., Chan T., McAuliffe S., Chenzbraun A. (2009). Non-rheumatic annular mitral stenosis: prevalence and characteristics. Euro. J. Echocardiogr..

[10] Iung B., Baron G., Butchart E.G., Delahaye F., Gohlke-Bärwolf C., Levang O.W., Tornos P., Vanoverschelde J.L., Vermeer F., Boersma E. (2003). A prospective survey of patients with valvular heart disease in Europe: the Euro Heart Survey on valvular heart disease. Eur. Heart J..

[11] Toufan M., Javadrashid R., Pak N., Gojazadeh M., Khalili M. (2012). Relationship between incidentally detected calcification of the mitral valve on 64-row multidetector computed tomography and mitral valve disease on echocardiography. Int. J. General Med..

[12] Benjamin E.J., Plehn J.F., D’Agostino R.B., Belanger A.J., Comai K., Fuller D.L., Wolf P.A., Levy D. (1992). Mitral annular calcifcation and the risk of stroke in an elderly cohort. New England J. Med..

[13] Nair C.K., Thomson W., Ryschon K., Cook C., Hee T.T., Sketch M.H. (1989). Long-term follow-up of patients with echocardiographically detected mitral anular calcium and comparison with age-and sex-matched control subjects. Am. J. Cardiol..

[14] Lip G.Y.H., Collet J.P., de Caterina R., Fauchier L., Lane D.A., Larsen T.B., Marin F., Morais J., Narasimhan C., Olshansky B. (2017). Antithrombotic therapy in atrial fibrillation associated with valvular heart disease: a joint consensus document from the European Heart Rhythm Association (EHRA) and European Society of Cardiology Working Group on Thrombosis, endorsed by the ESC Working. Europace.

[15] Karthikeyan G., Connolly S.J., Yusuf S. (2020). Overestimation of stroke risk in rheumatic mitral stenosis and the implications for oral anticoagulation. Circulation.

[16] Otto C.M., Nishimura R.A., Bonow R.O., Carabello B.A., Erwin J.P., Gentile F., Jneid H., Krieger E.V., Mack M., McLeod C. (2020). ACC/AHA guideline for the management of patients with valvular heart disease: a report of the American college of cardiology/American heart association joint committee on clinical practice guidelines. J. Am. Coll. Cardiol..

[17] A. Vahanian, F. Beyersdorf, F. Praz, M. Milojevic, S. Baldus, J. Bauersachs, D. Capodanno, L. Conradi, M. de Bonis, R. de Paulis et al., ESC/EACTS Guidelines for the management of valvular heart disease, Euro. Heart J. [Internet] (2021). <https://academic.oup.com/eurheartj/advance-article/doi/10.1093/eurheartj/ehab395/6358470>.

[18] Chao T.-F., Joung B., Takahashi Y., Lim T.W., Choi E.-K., Chan Y.-H., Guo Y., Sriratanasathavorn C., Oh S., Okumura K., Lip G.Y.H. (2022). 2021 Focused update consensus guidelines of the Asia pacific heart rhythm society on stroke prevention in atrial fibrillation: executive summary. Thromb. Haemost..

[19] Pengo V., Barbero F., Biasiolo A., Pegoraro C., Noventa F., Iliceto S. (2003). Prevention of thromboembolism in patients with mitral stenosis and associated atrial fibrillation: effectiveness of low intensity (INR target 2) oral anticoagulant treatment. Thromb. Haemost..

[20] Pérez-Gómez F., Salvador A., Zumalde J., Iriarte J.A., Berjón J., Alegría E., Almería C., Bover R., Herrera D., Fernández C. (2006). Effect of antithrombotic therapy in patients with mitral stenosis and atrial fibrillation: a sub-analysis of NASPEAF randomized trial. Eur. Heart J..

[21] Chiang C.-W., Lo S.-K., Ko Y.-S., Cheng N.-J., Lin P.J., Chang C.-H. (1998). Predictors of systemic embolism in mitral stenosis. Ann. Int. Med..

[22] De Caterina R., John Camm A. (2016). Non-Vitamin K antagonist oral anticoagulants in atrial fibrillation accompanying mitral stenosis: the concept for a trial. Europace..

[23] Lynge E., Sandegaard J.L., Rebolj M. (2011). The Danish national patient register. Scandinav. J. Public Health..

[24] Schmidt M., Pedersen L., Sørensen H.T. (2014). The danish civil registration system as a tool in epidemiology. Eur. J. Epidemiol..

[25] J. Sundbøll, K. Adelborg, T. Munch, T. Frøslev, H. Toft Sørensen, H. Erik Bøtker, M. Schmidt, Positive predictive value of cardiovascular diagnoses in the Danish National Patient Registry: a validation study. BMJ Open. [Internet] 6 (2016). <http://www.epidata.dk>. 10.1136/bmjopen-2016-012832 PMC5129042 27864249

[26] Wallach Kildemoes H., Toft Sørensen H., Hallas J. (2011). The Danish national prescription registry. Scandinav. J. Public Health..

[27] Friberg L., Skeppholm M., Terént A. (2015). Benefit of anticoagulation unlikely in patients with atrial fibrillation and a CHA 2 DS 2-VASc score of 1. J. Am. Coll. Cardiol..

[28] Pressman G.S., Ranjan R., Park D.H., Shim C.Y., Hong G.-R. (2020). Degenerative mitral stenosis versus rheumatic mitral stenosis. Am. J. Cardiol..

[29] Danish Society of Cardiology. National Kardiologisk Behandlingsvejledning [Internet]. 2021 [cited 2021 Dec 13]; Available from: nbv.cardio.dk.

[30] de Caterina R., Camm A.J. (2014). What is “valvular” atrial fibrillation? A reappraisal. Euro. Heart J..

[31] Karthikeyan G., Connolly S.J., Ntsekhe M., Benz A., Rangarajan S., Lewis G., Yun Y., Sharma S.K., Maklady F., Elghamrawy A.E., Kazmi K., Cabral T.T.J., Dayi H.u., Changsheng M.a., Gitura B.M., Avezum A., Zuhlke L., Lwabi P., Haileamlak A., Ogah O., Chillo P., Paniagua M., ElSayed A., Dans A., Gondwe-Chunda L., Molefe-Baikai O.J., Gonzalez-Hermosillo J.A., Hakim J., Damasceno A., Kamanzi E.R., Musuku J., Davletov K., Connolly K., Mayosi B.M., Yusuf S. (2020). The INVICTUS rheumatic heart disease research program: rationale, design and baseline characteristics of a randomized trial of rivaroxaban compared to vitamin K antagonists in rheumatic valvular disease and atrial fibrillation. Am. Heart J..

[32] Lühdorf P., Overvad K., Schmidt E.B., Johnsen S.P., Bach F.W. (2017). Predictive value of stroke discharge diagnoses in the Danish National Patient Register. Scandinav. J. Public Health.

[33] Krarup L.H., Boysen G., Janjua H., Prescott E., Truelsen T. (2007). Validity of stroke diagnoses in a national register of patients. Neuroepidemiology..

[34] Rix T.A., Riahi S., Overvad K., Lundbye-Christensen S., Schmidt E.B., Joensen A.M. (2012). Validity of the diagnoses atrial fibrillation and atrial flutter in a Danish patient registry. Scandinav. Cardiovasc. J..

