# Supplementary material for: Risk of stroke or systemic embolism in patients with degenerative mitral stenosis with or without atrial fibrillation: A cohort study
Source: Int J Cardiol Heart Vasc. 2022 Oct 7;43:101126. doi: 10.1016/j.ijcha.2022.101126 (PMC9550603; doi:10.1016/j.ijcha.2022.101126)
Supplement: Supplementary data 1 [file mmc1.docx]

| **Supplemental Table 1:** Variables and data sources | | |
| --- | --- | --- |
| **Variable** | **ICD-10 code(s)** | **Other – ATC- or SKS code(s)** |
| Degenerative mitral stenosis | I342 |  |
| Atrial fibrillation | I48 |  |
| Ischemic stroke | I63 I64 |  |
| Systemic embolism | I74 K550 N280 D735 |  |
| Hypertension (1 ICD-10 code and minimum 2 ATC codes) | I10 I11 I12 I13 I15 | C09BB04 C09DA C09DB C09DX01 C09DX04 C07B |
| Diabetes mellitus | E10 E11 E14 |  |
| Dyslipidemia | E78 |  |
| Congestive heart failure | I110 I130 I132 I50 |  |
| Ischemic heart disease | I20 I21 I23 I24 I25 |  |
| Peripheral artery disease | I702 I739A I739C I74 |  |
| Chronic kidney disease | E102 E112 E142 I120 I131 I132 I150 I151 N03 N05 N06 N07 N08 N110 N14 N15 N16 N18 N19 N26 N27 N280 N391 Q61 |  |
| Cardiomyopathy | I420 I421 I422 I429 |  |
| Obesity | E65 E66 |  |
| CHA_2_DS_2_-VASc score components | I110 I130 I132 I420 I50 I501 I509 E10 E11 E12 E13 E14 H360 O240 O241 O242 O243 I63 I64 G45 I74 K550 N280 D735 I21 I23 I702 I703 I704 I705 I706 I707 I708 I709 I71 I739 I700 |  |
| Mitral valve surgery |  | KFK |
| Aortic valve surgery |  | KFM |
| Acquired aortic valve disease | I35 I06 |  |
| Congenital aortic- and mitral valve disease | DQ23 |  |
| Mitral valve prolapse | I341 |  |
| Rheumatic mitral valve disease | I05 I080A I081A I083A |  |
| Warfarin |  | B01AA03 |
| Phenprocoumon |  | B01AA04 |
| Aspirin |  | B01AC06 |
| Thienopyridines (clopidogrel, ticagrelor, prasugrel) |  | B01AC04 B01AC24 B01AC22 |
| Calcium-channel blockers |  | C07F C08 C09BB C09DB |
| Angiotensin-converting enzyme inhibitor/angiotensin receptor blocker |  | C09 |
| Beta-blockers |  | C07 |
| Diuretics |  | C03C C03EB C02DA C02L C03A C03B C03D C03EA C03X C07C C07D C08G C09BA C09DA C09XA52 |
| Statins |  | C10 |
| Percutaneous coronary intervention |  | KFNG |
| Coronary artery bypass graft |  | KFNA KFNC KFND KFNE |
| Pacemaker |  | BFCA0 BFCA6 BFCA9 |
| Ablation therapy |  | BFFB |
| Dialysis |  | BJFD2 |
