# Supplementary material for: Risk of stroke or systemic embolism in patients with degenerative mitral stenosis with or without atrial fibrillation: A cohort study
Source: Int J Cardiol Heart Vasc. 2022 Oct 7;43:101126. doi: 10.1016/j.ijcha.2022.101126 (PMC9550603; doi:10.1016/j.ijcha.2022.101126)
Supplement: Supplementary data 2 [file mmc2.docx]

|  | **Supplementary Table 2:** Events and incidence rates per 100 person-years (95% CI) for ischemic stroke or systemic embolism and all-cause mortality after 1 year of follow-up (with 10-day quarantine period) | | | | |  |
| --- | --- | --- | --- | --- | --- | --- |
|  | | Ischemic stroke or systemic embolism | | All-cause mortality | |  |
|  |  | No. of events | Incidence rate (95% CI) | No. of events | Incidence rate (95% CI) | |
| DMS without AF group  (n = 741) | 43 | 6.65 (4.93 – 8.97) | 105 | 15.66 (12.93 – 18.96) | |  |
| DMS with AF group  (n = 421) | 20 | 5.77 (3.72 – 8.94) | 82 | 23.06 (18.57 – 28.63) | |  |

AF = atrial fibrillation, CI = confidence interval, DMS = degenerative mitral stenosis, n = number of patients
