# Supplementary material for: Risk of stroke or systemic embolism in patients with degenerative mitral stenosis with or without atrial fibrillation: A cohort study
Source: Int J Cardiol Heart Vasc. 2022 Oct 7;43:101126. doi: 10.1016/j.ijcha.2022.101126 (PMC9550603; doi:10.1016/j.ijcha.2022.101126)
Supplement: Supplementary data 3 [file mmc3.docx]

|  | **Supplementary Table 3**: Events and incidence rates per 100 person-years (95% CI) for ischemic stroke or systemic embolism and all-cause mortality after 1 year of follow-up stratified by prior thromboembolic event (with 10-day quarantine period) | | | |
| --- | --- | --- | --- | --- |
|  | Ischemic stroke or systemic embolism | | All-cause mortality | |
|  | No. of events | Incidence rate (95% CI) | No. of events | Incidence rate (95% CI) |
| DMS without AF group   - No prior thromboembolic event (n = 659) | 27 | 4.64 (3.18 – 6.76) | 98 | 16.51 (13.55 – 20.13) |
| - Prior thromboembolic event (n = 82) | 16 | 24.93 (15.27 – 40.69) | 7 | 9.09 (4.34 – 19.08 |
| DMS with AF group   - No prior thromboembolic event (n = 370) | 15 | 4.82 (2.91 – 8.00) | 68 | 21.44 (16.90 – 27.19) |
| - Prior thromboembolic event (n = 51) | 5 | 13.95 (5.81 – 33.51) | 14 | 36.44 (21.58 – 61.53) |

AF = atrial fibrillation, CI = confidence interval, DMS = degenerative mitral stenosis, n = number of patients.
