# Supplementary material for: Risk of stroke or systemic embolism in patients with degenerative mitral stenosis with or without atrial fibrillation: A cohort study
Source: Int J Cardiol Heart Vasc. 2022 Oct 7;43:101126. doi: 10.1016/j.ijcha.2022.101126 (PMC9550603; doi:10.1016/j.ijcha.2022.101126)
Supplement: Supplementary data 4 [file mmc4.docx]

| **Supplemental Table 4**: Events and incidence rates per 100 person-years (95% CI) for AF in patients with DMS without AF at baseline after 1 year of follow-up | |
| --- | --- |
| No. of events | Incidence rate (95% CI) |
| 96 | 15.66 (12.82 – 19.12) |

AF = atrial fibrillation, CI = confidence interval, DMS = degenerative mitral stenosis
