# Supplementary material for: Risk of stroke or systemic embolism in patients with degenerative mitral stenosis with or without atrial fibrillation: A cohort study
Source: Int J Cardiol Heart Vasc. 2022 Oct 7;43:101126. doi: 10.1016/j.ijcha.2022.101126 (PMC9550603; doi:10.1016/j.ijcha.2022.101126)
Supplement: Supplementary data 5 [file mmc5.docx]

| **Supplemental Table 5**: Events and incidence rates per 100 person-years (95% CI) after 1 year of follow-up for stroke or systemic embolism in patients not treated with OAC at index date, censored for when OAC was started during follow-up | |
| --- | --- |
| No. of events | Incidence rate (95% CI) |
| 67.32 | 7.30 (5.49 – 9.91) |

AF = atrial fibrillation, CI = confidence interval, DMS = degenerative mitral stenosis, OAC = oral anticoagulants
