# Supplementary figures and images for: Risk of stroke or systemic embolism in patients with degenerative mitral stenosis with or without atrial fibrillation: A cohort study
Source: Int J Cardiol Heart Vasc. 2022 Oct 7;43:101126. doi: 10.1016/j.ijcha.2022.101126 (PMC9550603; doi:10.1016/j.ijcha.2022.101126)

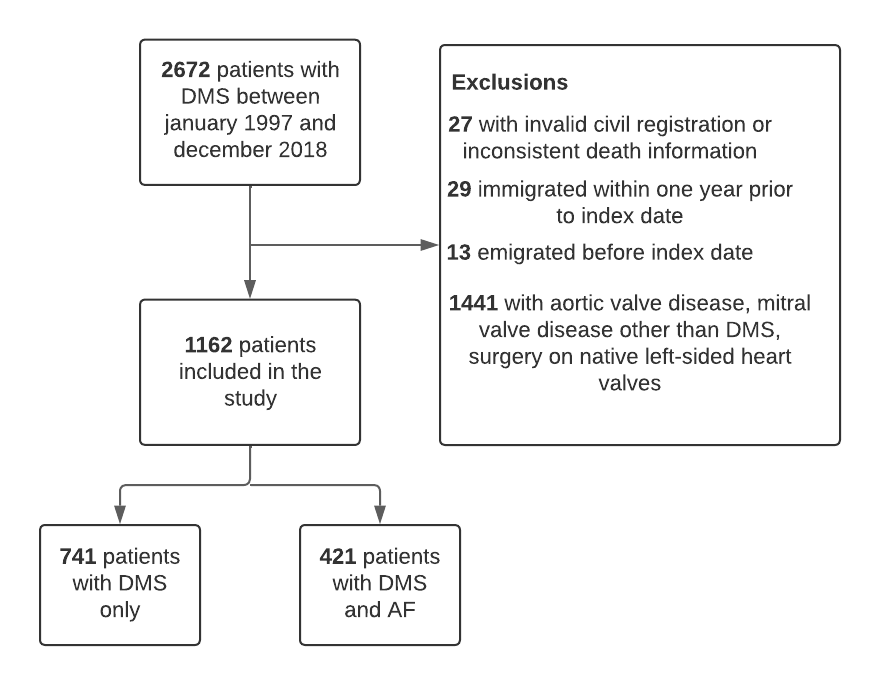

Supplement: Supplementary data 6 [file mmc6.zip › Supplemental figure 1.png.png]
